# Synthesis of 1,2-Disubstituted Adamantane Derivatives by Construction of the Adamantane Framework †

**DOI:** 10.3390/molecules28227636

**Published:** 2023-11-16

**Authors:** Matthew Todd, Radim Hrdina

**Affiliations:** Department of Organic Chemistry, Faculty of Science, Charles University, Hlavova 8, 12043 Praha, Czech Republic; toddm@natur.cuni.cz

**Keywords:** adamantane, noradamantane, protoadamantane, homoadamantane, diamondoids, rearrangement, total synthesis, alkyl shifts, ring contraction, ring expansion

## Abstract

This review summarizes achievements in the synthesis of 1,2-disubstituted adamantane derivatives by the construction of the tricyclic framework either by total synthesis or by ring expansion/contraction reactions of corresponding adamantane homologues. It is intended to complement reviews focusing on the preparation of 1,2-disubstituted derivatives by C–H functionalization methods.

## 1. Introduction

This year, we celebrate 90 years of adamantane chemistry, since the first isolation of the molecule from Hodonín crude oil and its structural elucidation at the University of Chemistry and Technology in Prague [1]. Since then, this tricyclic molecule has appeared as a building block in organic synthesis alongside other common aliphatic compounds [2]. Many reviews have already summarized original works describing its first preparation [3], industrial synthesis [4], properties [5], reactivity and functionalization [6], and applications of its derivatives in medicine [7,8,9,10,11,12], catalysis [13], material science [14,15,16,17], and other fields. Compounds with a repeating adamantane unit are sometimes called diamondoids (after the diamond crystal lattice) [18,19] and are also extracted from crude oil [20]. Diamantane can be effectively synthesized [21], while higher diamondoids such as triamantane, tetramantane, etc., are typically separated from petroleum by column chromatography [22]. As for any other alkane, the first functionalization of adamantane or its congeners is based on the oxidation of the C–H bond to a selected functional group [23]. This group then influences the second functionalization step, either electronically in non-directed reactions [24,25,26] or as a directing group in directed C–H functionalization reactions [27]. This is not the only way to access disubstituted adamantane derivatives. The adamantane skeleton can be constructed from bicyclic precursors or by ring expansion and contraction reactions from suitable starting materials, which is the topic of this review.

## 2. 1,2-Disubstituted Adamantane Derivatives

The adamantane skeleton is often used as a building block when steric bulk, lipophilicity, or rigidity is required [28]. Formally replacing one hydrogen atom at carbon 1 and one hydrogen atom at carbon 2 in the adamantane molecule with two substituents (the same or different) leads to the formation of compounds that are chiral [29]. To date, mostly 1,3-disubstituted achiral adamantane derivatives, utilized as rigid spacers, have been reported. The properties of chiral derivatives can then be exploited in applications where chirality plays a role, such as enantioselective catalysis [30,31,32], the synthesis of enantiopure compounds [33,34], interactions of enantiopure compounds with material [35,36] or light [37], and other physical, chemical, or biological applications [38,39,40]. The low stability of the adamant-1-ene molecule, which has a rather biradical character, does not allow the use of classical alkene chemistry for the formation of 1,2-disubstituted adamantane compounds [41,42,43]. The 1,2-substitution pattern has to be accessed in a different way, either by directed C–H functionalization reactions [27] or by the construction of an adamantane scaffold.

### 2.1. Synthesis of 1,2-Disubstituted Adamantane Derivatives by Total Synthesis from Acyclic, Monocyclic, or Bicyclic Starting Materials

**Scheme 1:**
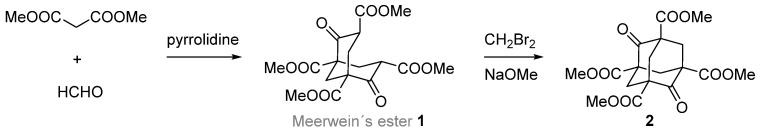
First synthesis of substituted adamantane scaffold from simple acyclic starting materials.

The first construction of the adamantane molecule from acyclic starting materials, namely formaldehyde and dimethyl malonate, includes the formation of intermediate **1**, which belongs to the reviewed class of compounds (Scheme 1) [3,44].

During the surge in adamantane chemistry in the 1960s, it became apparent that its chemical reactivity limits the access to densely substituted derivatives directly from its parent hydrocarbon and especially in the secondary bridge positions [2]. Constructing the adamantane core ‘ground-up’, starting from substituted monocyclic compounds, appeared to be a fast and promising strategy to circumvent this issue.

**Scheme 2:**
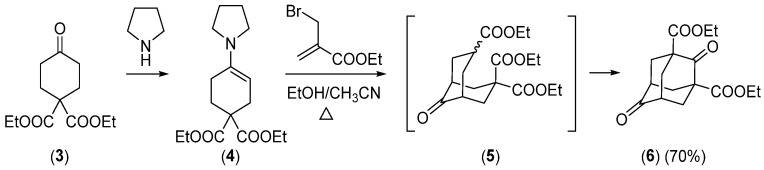
Enamine (**4**) condensation with ethyl 2-(bromoethyl)acrylate leads to the formation of the intermediate bicycle **5**, which undergoes intramolecular condensation to give the adamantane derivative **6**.

In the next published example of this approach (Scheme 2), the monocyclic compound **3** was combined with pyrrolidine to form the corresponding enamine **4**. At reflux, the Michael addition of enamine **4** to 2-(bromoethyl)acrylate, followed by the regeneration of the enamine on the opposite side, avoiding allylic strain, and displacement of the bromine resulted in the formation of the bicyclic intermediate **5**, which undergoes Dieckmann condensation to give the densely substituted adamantane core **6** in a 70% yield [45].

**Scheme 3:**
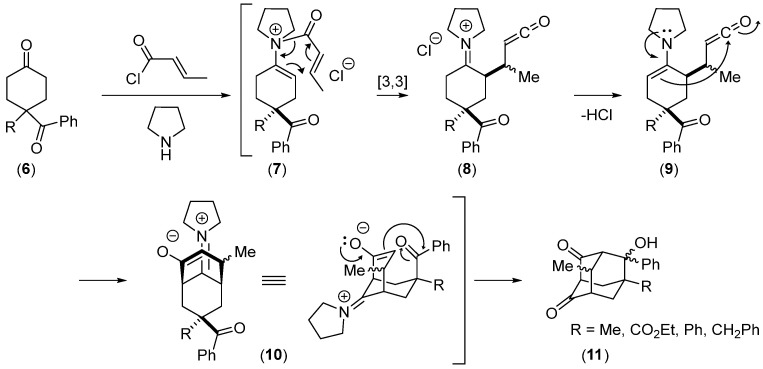
Condensation of enamine **7** with crotonoyl chloride leads to intermediate **10** that, upon cyclization, gives products **11**.

Inspired by the previous example, more syntheses were carried out, varying the monocyclic compounds, formed enamines, Michael acceptors, and conditions, to explore additional possibilities of the transformation. Extensive investigation of the reaction mechanism was carried out to rationalize the diastereoselectivity of the process (Scheme 3). Upon enamine formation from diketone **6** and pyrrolidine, the following step is proposed to be *N*-acylation by crotonoyl chloride. An intramolecular [3,3] sigmatropic rearrangement of intermediate **7** proceeds through a transition state whereby the formed substituent (alkylketene) has a *cis*-geometric relationship with the benzoyl substituent due to the attractive electronic interactions of these two groups and the steric hindrance of the R substituent. The absence of a di-one in which the carbonyl group occupies the equatorial position is in agreement with the author’s proposal. The intermediate ketene **8** reforms its enamine **9**, which cyclizes to give the corresponding enolate **10**, which undergoes further cyclization, forming the products **11** in yields ranging from 30 to 50% [46,47,48,49].

**Scheme 4:**
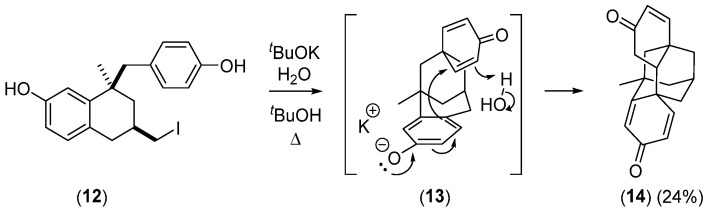
Base-promoted cyclization of starting material **12** gives the bicyclic intermediate **13** with a final Michael addition closing the adamantane scaffold **14**.

An alternative approach is depicted in Scheme 4. Starting from the diphenol **12** at high temperatures in a sealed tube, deprotonation with *tert*-butoxide generates the phenoxide acting as a C-nucleophile, which effects a ring-closing attack at the *cis*-disposed iodomethyl group, generating the bicyclic intermediate **13**. Subsequent intramolecular Michael addition of the phenolate moiety to the dienone system gives rise to the adamantane scaffold **14** in a 24% yield. The relatively low yield is attributed to the fact that the *cis*-isomer **12** is actually the minor component of the starting material, which is used as a mixture of isomers in a ratio of 3:1. The authors also report that the excess of base results in the isolation of intermediate **13** exclusively [50].

**Scheme 5:**
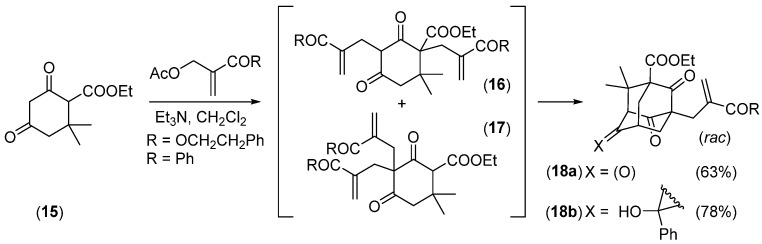
Cascade condensation reactions of starting material **15** with various acrylates (or enones) give products **18a** and **18b**.

The rather facile synthesis of a densely substituted adamantane core is demonstrated in a simple one-pot reaction of di-one **15** with an excess of acrylates (or enones) and triethylamine that proceeds through a cascade of aldol, Michael, and Dieckmann condensation, giving the products **18a** and **18b** in high yields (Scheme 5). The yield declines when the procedure is carried out stepwise and the intermediates **16** and **17** are isolated. The reaction proved to be sensitive to the steric bulk of the acrylate (or enone) R group. With R = Ph, the reaction progresses readily at room temperature, whereas R = OCH_2_CH_2_Ph requires higher temperatures. No formation of the product was observed for R = *^t^*BuO [51].

**Scheme 6:**
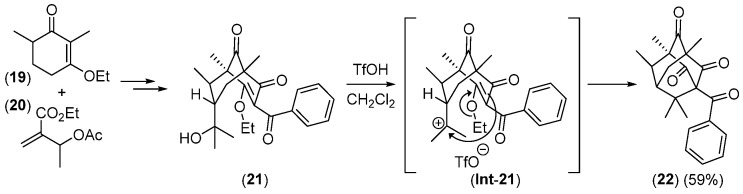
Successive Michael additions and further modifications formed the bicyclic compound **21** that undergoes acid-catalyzed cyclization to form the product **22**.

The authors of the next example (Scheme 6) started by combining the monocyclic starting material **19** and acrylate **20**, which undergo a series of Michael additions, forming a bicyclic core that was further modified to compound **21**. After screening various Brønsted acids and conditions, the optimal result was obtained by refluxing the bicyclic compound **21** in dichloromethane with triflic acid. The acid promoted dehydration to intermediate **Int**-**21** and the dealkylation of the enol ether formed a carbon–carbon bond, furnishing the adamantane scaffold **22** in a 59% yield [52].

**Scheme 7:**
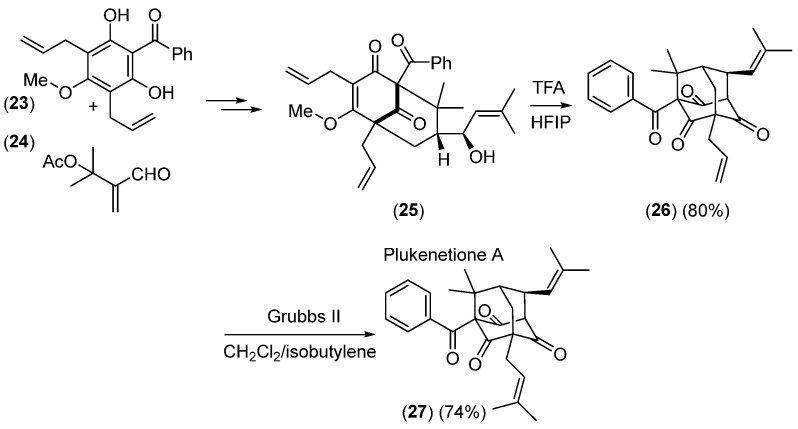
Acid-catalyzed cyclization of bicyclic compound **25** to form the adamantane core **26** followed by metathesis, which is the final step in the total synthesis of Plukenetione A (**27**).

A similar approach was applied in the total synthesis of Plukenetione A, a natural product containing an adamantane core (Scheme 7). A series of acid- and base-catalyzed condensation and cyclization steps of the starting material **23** and enal **24** afforded the bicyclic compound **25**, which was subjected to acid-promoted cyclization between the enol ether moiety and the carbocation generated from the free hydroxyl group to form the product **26**. The final metathesis step with isobutylene and a Grubbs catalyst gave the desired Plukenetione A (**27**) [53].

### 2.2. Synthesis of 1,2-Disubstituted Adamantane Derivatives from Bicyclic Starting Materials

The second effective approach to 1,2-disubstituted adamantane compounds utilizes the cyclization of bicyclic precursors, namely derivatives of bicyclo[3.3.1]nonanes, which can be obtained from simple building blocks (as shown previously) or through the ring opening of readily available 1,3-disubstituted adamantane derivatives [54,55].

#### 2.2.1. Diolefines as Precursors

**Scheme 8:**
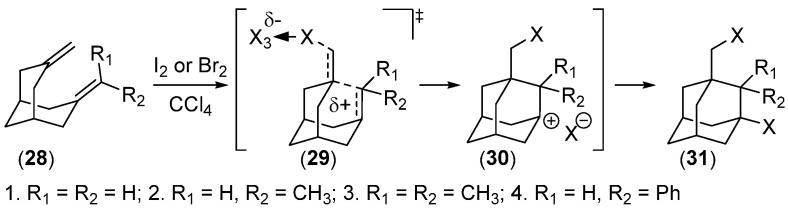
Reaction of diolefines **28** with bromine or iodine led to the formation of products **31**.

Bicyclo[3.3.1]nonane-derived diolefines are a class of compounds that have been demonstrated to undergo ring closure to the adamantane scaffold. In the first example (Scheme 8), the diolefines **28** were used as a platform to study the mechanism of transannular cyclization reactions. The exposure of starting materials **28** to bromine or iodine forms a charge transfer complex **29** by attacking the less substituted methylene group, due to stereo-electronic factors, followed by synchronous cyclization to form the stable 1-adamantyl cation ion pair **30**, leading to the products **31**. Reactions were not carried out for preparative purposes; therefore, no yields were reported [56,57].

**Scheme 9:**
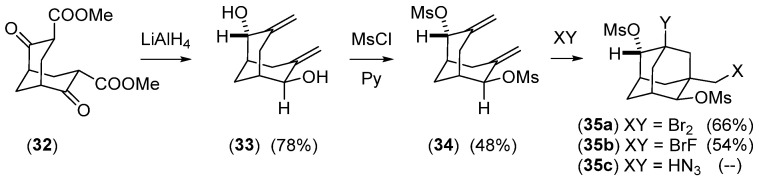
Diolefine **34** cyclizes to form products **35a** and **35b**.

The next example was carried out for synthetic purposes on a larger scale. The starting material **32** (Scheme 9) was readily obtained from the partially decarboxylated Meerwein’s ester **1**, which was reduced to the corresponding diol **33** and subsequently protected. The exposure of **34** to bromine successfully afforded the dibromo derivative **35a** as the major product in a yield of 66%. Halogenation with the mixed halogen BrF gave the product **35b** in a yield of 54%. Attempts to implement a nitrogen functionality (**35c**) by in situ generated HN_3_ failed, resulting in a mixture of side products due to the reaction of the solvent with the intermediate 1-adamantyl carbocation [58].

**Scheme 10:**
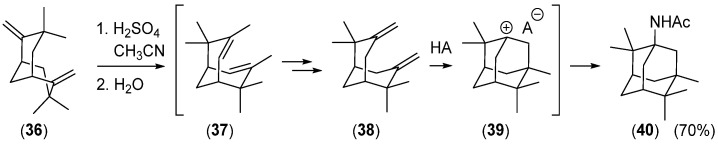
Ritter reaction of diolefine **36** provides acetamide **40**.

The nitrogen functional group was successfully introduced under Ritter conditions (Scheme 10). The starting material **36** undergoes a series of acid-catalyzed Wagner–Meerwein rearrangements of the geminal methyl groups, eliminations, and isomerizations of *endo*-cyclic to *exo*-cyclic double bonds, generating diolefine **38**. Cyclization leads to the stable tertiary 1-adamantyl cation **39**, which is trapped by acetonitrile with the presence of water, giving the final acetamide **40** in 70%. Under milder Ritter conditions, the reaction halted at diolefine **37**, which was retrieved in a yield of 75% and could be transformed to the product **40**, in agreement with the proposed mechanistic pathway [59].

**Scheme 11:**
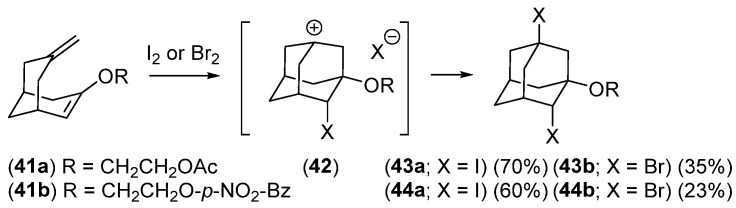
Exposure of enol ethers **41a** and **41b** to iodine or bromine leads to halogenated adamantanes **43ab** and **44ab**.

Enol ether derivatives **41a** and **41b** (Scheme 11) of bicyclo[3.3.1]nonanes exposed to iodine and bromine proceed through electrophilic addition to the more electron-rich π-system, triggering cyclization towards the intermediate ion pair **42** that results in products **43ab** and **44ab**. The transformation with iodine gave higher yields (70%) compared to the bromination products (35%) [60].

**Scheme 12:**
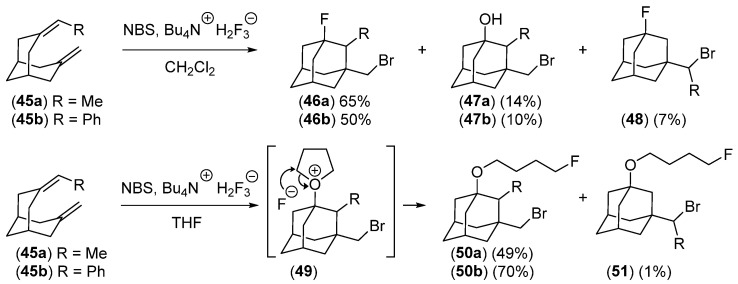
Halo-fluorination of **45ab** gives bromo-fluoro derivatives **46ab** and **47ab** in CH_2_Cl_2_ and fluoro-alkyl ethers **50ab** in THF.

Halo-fluorination was also performed for the preparation of mixed halo-adamantane derivatives (Scheme 12). *N*-bromosuccinimide (NBS) provides the electrophilic bromine that triggers the cyclization of diolefines **45ab** on the less substituted double bond, with a nucleophilic fluorine atom being provided by tetrabutyl ammonium dihydrogen trifluoride to trap the cationic intermediate. Bromo-fluoro compounds **46a** and **46b** were the major products, with side products **47a** and **47b**, resulting from the addition of water to the cationic intermediate; in the case of starting material **45b** (R = Ph), a small amount of side product **48** occurred, resulting from the bromonium cation being formed on the R-substituted methylene group.

When the reaction was carried out in tetrahydrofuran (THF) as the solvent, its participation, trapping the 1-adamantyl cation, was observed to form the intermediate oxonium ion **49** with subsequent ring opening by the nucleophilic fluoride, giving the fluoro-alkyl ethers **50ab** instead. Reactions in tetrahydropyran (six-membered ether) led to the fluorinated products **46ab** and **47ab**, whereas oxetane (four-membered) provided a shorter ether linkage. In ethylene oxide (three-membered), the reaction gave a mixture of bromo- and fluoro-alkyl ethers in smaller yields [61].

**Scheme 13:**
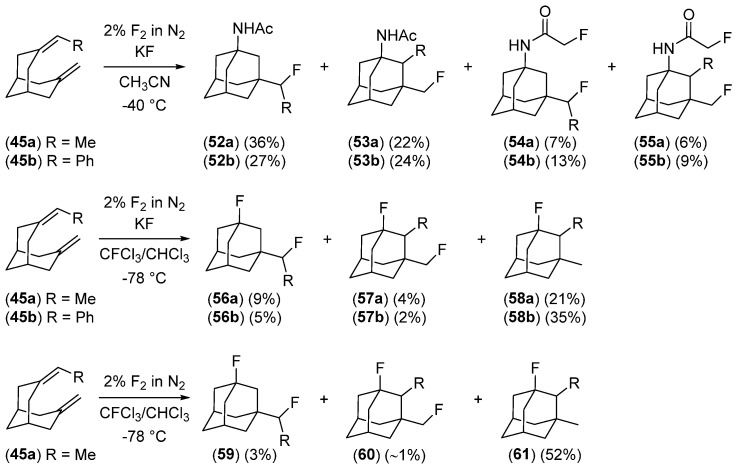
Synthesis of variously substituted adamantane derivatives by elemental fluorination.

The addition of elemental fluorine (F_2_) to unsaturated systems can either proceed as a radical process or has an electrophilic ionic nature (Scheme 13). Both pathways would leave a ‘footprint’ in product distributions starting from **45ab**, where a radical process would favor noradamantyl derivatives, in contrast to the electrophilic ionic pathway preferring the transannular cyclization towards adamantyl cations, as predicted by computational studies [62].

The absence of even trace amounts of noradamantane derivatives, thus strictly favoring the adamantyl cation formation, is in strong suggestion of the ionic electrophilic nature of the reaction progress. Compared to uncharged sources of electrophilic fluorine, the (non)selectivity for substituted and unsubstituted double bonds is typical for a charged electrophilic species and in sharp contrast to Selectfluor^®^, which strongly prefers the substituted double bonds [63]. Additions were studied at cryogenic temperatures in both nucleophilic and non-nucleophilic solvents.

Major products **58ab** and **61** in the CFCl_3_/CHCl_3_ solvent system are formed due to protic acid-triggered cyclization by HF, formed from a side reaction of F_2_ and CHCl_3_. Reactions in pure CFCl_3_ resulted in poor yields and tarry complex mixtures of products. Side products **56ab**, **57ab**, **59**, and **60** were the result of the electrophilic fluorine-triggered cyclization. The addition of KF provided a nucleophilic fluorine source to suppress the trapping of the cationic intermediates by side nucleophiles.

Reactions in wet acetonitrile successfully trapped the cationic intermediates, forming the corresponding acetamides **52ab** and **53ab**. Additional fluorination also resulted in the formation of side products **54ab** and **55ab** [62].

#### 2.2.2. Ketoolefines as Precursors

**Scheme 14:**
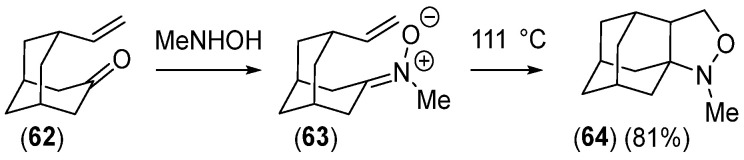
The 1,3-dipolar cycloaddition of nitrone **63** leads to product **64**.

Ketoolefines derived from bicyclo[3.3.1]nonanes are versatile starting materials for the building of substituted adamantane cores. The condensation of ketoolefine **62** (Scheme 14) with *N*-methyl hydroxylamine forms the corresponding nitrone **63**. Elevated temperatures are necessary for the equilibration of E/Z nitrone configurations in order for the 1,3-dipolar cycloaddition to proceed. Ring formation afforded the annulated adamantane product **64** in a high yield (81%), which can be easily transformed through catalytic hydrogenation into its amino-alcohol. Although only the *N*-methyl-substituted hydroxylamine was investigated, the authors mentioned that the yields of other substrates were not much affected by the alkyl groups’ nature [64].

**Scheme 15:**
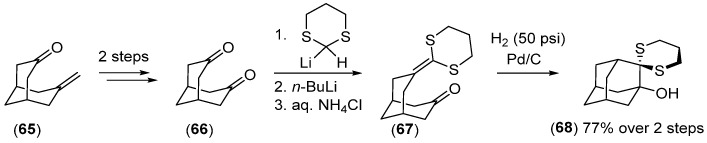
Reductive cyclization of mono ketene dithioacetal **67** gave **68** as the product.

The ketoolefine **65** was converted into the di-one **66** (Scheme 15). The following reaction with 2-lithio-1,3-dithiane and subsequent treatment with *n*-BuLi gave the addition-elimination product **67**. Catalytic hydrogenation under increased pressure resulted in the ring closure product **68** in a yield of 77% over two steps. Carrying out the procedure with flexible linear chain di-ones was unsuccessful. The spatial proximity of the two carbonyl groups was deemed as the key factor in the bond formation [65].

**Scheme 16:**
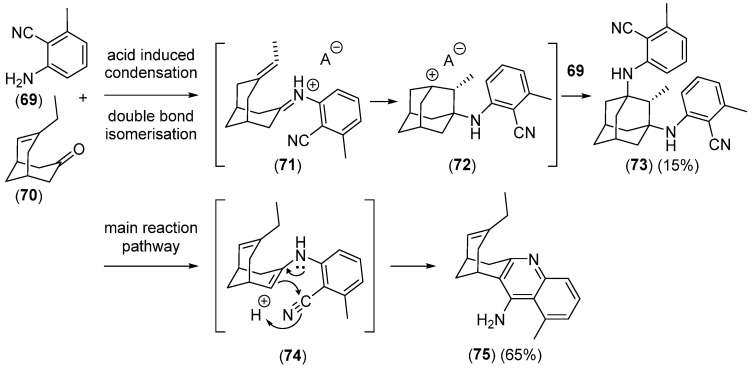
Compound **73** formed as a side product during the synthesis of **75**.

Compound **73** (Scheme 16) was obtained as a side product in a yield of 15% during the synthesis of **75**. The condensation of cyanoaniline **69** with ketoolefine **70** and the acid-catalyzed isomerization of the *endo*- to *exo*-cyclic double bond gave the iminium salt **71**. Transannular cyclization led to the 1-adamantyl cation intermediate **72** that was trapped by a second equivalent of cyanoaniline **69** to form the final product **73**. The main reaction pathway avoids double bond isomerization and forms the enamine **74**, which attacks the nitrile group. Aromatization gave a 65% yield of the major quinazoline-derived product **75** [66].

**Scheme 17:**
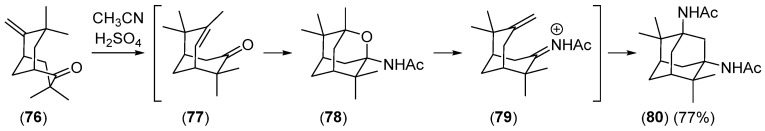
Ritter reaction of **76** leads to the bis-acetamide **80**.

A similar example to the reaction is described in Scheme 10. When subjecting the ketoolefine **76** (Scheme 17) to Ritter conditions, it undergoes a series of Wagner–Meerwein and Meinwald rearrangements driven by the release of transannular strain to afford the intermediate **77**. The acid-promoted cyclization of the double bond with the carbonyl oxygen generates a 2-oxa-1-adamantyl cation being trapped by acetonitrile that hydrolyzes to the hemiaminal **78**. Cleavage of the hemiaminal and dehydration leads to the familiar bicyclic iminium salt **79**, which cyclizes to the tertiary 1-adamantyl cation. The addition of acetonitrile and hydrolysis affords the final bis-acetamide **80** in a high yield of 77%. Varying the reaction times and severity of the reaction conditions, the authors were able to stop the reaction and isolate and characterize the individual intermediates (**77** and **78**) in moderate to good yields, gaining evidence for the proposed mechanism [67].

**Scheme 18:**
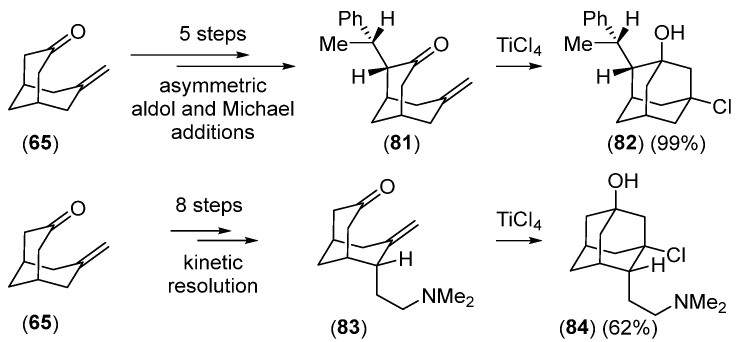
Synthesis of enantiomerically pure substituted adamantane derivatives **82** and **84**.

All 1,2-disubstituted adamantane derivatives are chiral. A synthetic pathway to enantiomerically pure derivatives is sought after in various applications. The common starting material **65** was converted through a series of asymmetric reactions and enantiomeric resolution steps into enantiomerically pure ketoolefines **81** and **83** (Scheme 18). Cyclization using titanium(IV) chloride retained their stereochemistry and gave both enantiomerically pure products **82** and **84** in good to high yields [68].

**Scheme 19:**
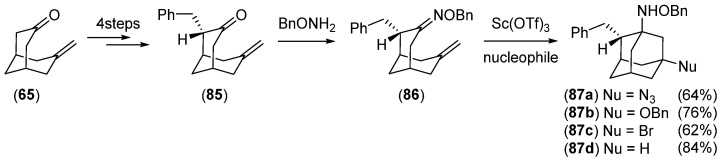
Lewis-promoted (or catalyzed) reaction of protected oxime **86** with various nucleophiles gives amino-adamantanes **87a–d**.

Using a similar strategy (Scheme 18), ketoolefine **65** was converted into starting material **85** (Scheme 19). Simple condensation with *O*-benzyl-hydroxylamine gave the benzyloxime **86**. Scandium(III) triflate initiated the cyclization step towards the 1-adamantyl cation that was trapped with various nucleophiles, providing products **87a–d** with yields ranging from 62 to 84%. Unprotected oximes, derived from hydroxylamine, gave noticeably lower yields. Stoichiometric amounts of the Lewis acid are needed for high yields, but it is possible to carry out the reaction with catalytic amounts (20 mol%) at the expense of slightly lower yields. The final products **87a–d** can be readily deprotected by hydrogen on palladized charcoal or zinc in acetic acid [69].

#### 2.2.3. Other Bicyclic Precursors

**Scheme 20:**
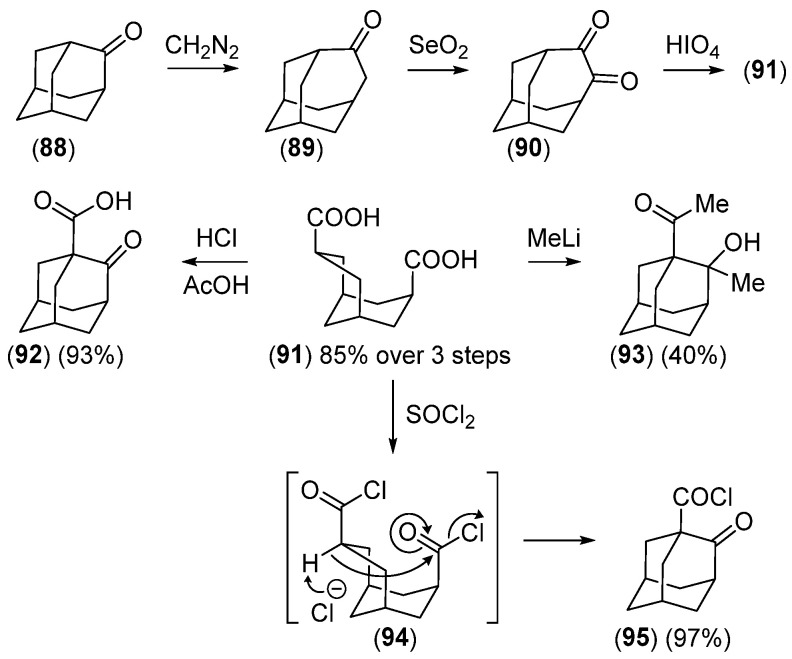
Di-carboxylic acid **91** can be converted to 1,2-disubstituted adamantanes **92**, **93**, and **95**.

Apart from diolefines and ketoolefines, other bicyclo[3.3.1]nonane derivatives have been used to build adamantane scaffolds. Starting from readily available adamantan-2-one (**88**) (Scheme 20), the Demjanov reaction expands it to homoadamantan-2-one **89**. Selenium oxide α-oxidation gives the di-one **90**, which is cleaved with periodic acid to afford dicarboxylic acid **91** in a high yield over three steps. Boiling the starting material **91** with thionyl chloride results in ring closure due to the close proximity of the two carbonyl groups (**94**), producing the acyl chloride **95** in a near quantitative yield. Additionally, the treatment of **91** with HCl or MeLi also gave the corresponding 1,2-disubstituted products **92** and **93**. The methyl ester of **91** was shown to form the methyl ester of **92** in a basic medium [70].

**Scheme 21:**
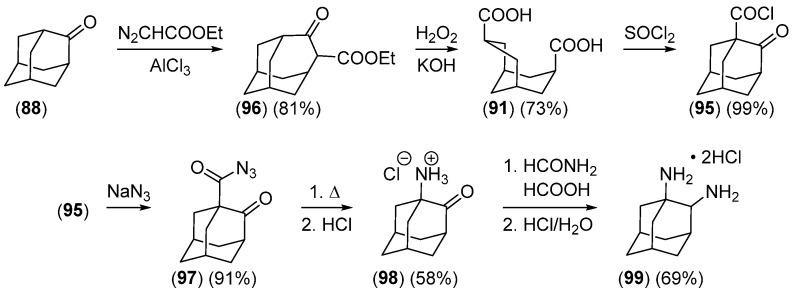
Multistep synthesis of 1,2-diamino adamantane (**99**).

The di-carboxylic acid **91** from adamantan-2-one (**88**) was also prepared through a Lewis acid-promoted Demjanov reaction with ethyl diazoacetate (**96**) and subsequent cleavage with alkaline hydrogen peroxide (Scheme 21) [71]. Reacting **91** with boiling thionyl chloride gave the acyl chloride **95** in a quantitative yield. Displacement of the chloride with sodium azide formed azide **97** in a high yield. The thermal decomposition of **97** led to the Curtius rearrangement. The intermediate isocyanate was exposed to HCl to give the hydrochloride salt **98**. Refluxing **98** with formamide and formic acid (Leuckart–Wallach reaction) resulted in the reductive amidation/hydrolysis product, obtaining the 1,2-diamine hydrochloride salt **99** in a high yield. The final racemic product was resolved into individual enantiomers and used for the synthesis of various chiral ligands. The power of the approach is that all steps were carried out without the necessity of column chromatography purification as the crystalline products were easily precipitated/recrystallized [29].

**Scheme 22:**
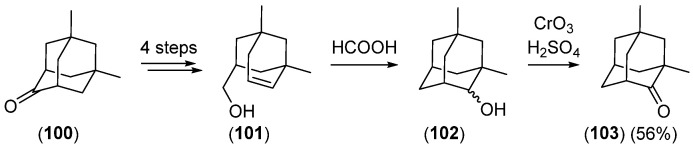
Acid-catalyzed rearrangement of primary alcohol **101** to secondary alcohol **102** with the adamantane framework, which was further oxidized to ketone **103**.

Primary alcohol **101** (Scheme 22) was subjected to an acid-catalyzed sigmatropic rearrangement when refluxed in formic acid to form the adamantane derivative **102**, which was further oxidized without purification using Jones reagent to ketone **103** in a 56% yield [72].

**Scheme 23:**
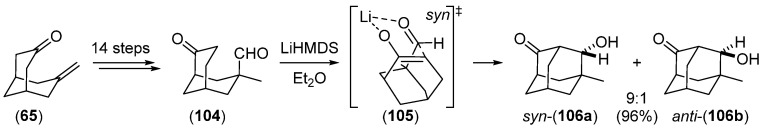
Ring closure of **104** under basic conditions gives a mixture of *syn*/*anti* products **106ab**.

As part of the study of aldol condensation transition states, the bicyclic keto-aldehyde **104** (Scheme 23) was treated with various bases in different solvents. For synthetic purposes (yield and selectivity), the best results were obtained using lithium hexamethyl disilazane (LiHMDS) in diethyl ether, with the *syn* keto-alcohol **106a** being the major product [73].

### 2.3. Synthesis of 1,2-Disubstituted Adamantane Derivatives by Ring Expansion Reactions

**Figure 1:**
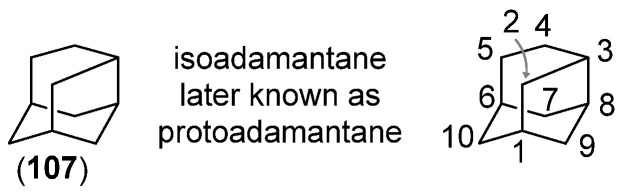
Protoadamantane (**107**).

**Figure 2:**
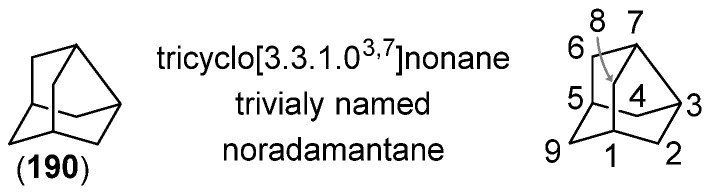
Noradamantane (**190**).

Adamantane represents the bottom of a thermodynamic stability well of C_10_H_16_ hydrocarbon constitutional isomers, where all others tend to fall to under thermodynamic conditions, already apparent from its method of industrial synthesis [4,74]. Close ring-contracted derivatives, such as protoadamantane (Figure 1) [75,76] and noradamantane (Figure 2) [77,78], have a certain degree of strain and have been used as ‘spring-loaded’ compounds that rearrange to more stable adamantane, revealing carbocations during the process. These reactions have been exploited in the synthesis of 1,2-disubstituted adamantane derivatives.

#### 2.3.1. The Protoadamantane Route

**Scheme 24:**
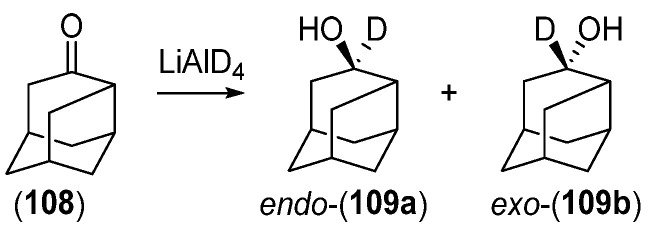
Reduction of protoadamantan-4-one (**108**) with LiAlD_4_.

The reduction of protoadamantan-4-one (**108**) with lithium aluminum deuteride gives a mixture of *endo*-(**109a**) and *exo*-protoadamantan-4-ol-*d*_4_ (**109b**) isomers that differ in reactivity (Scheme 24) [79].

**Scheme 25:**
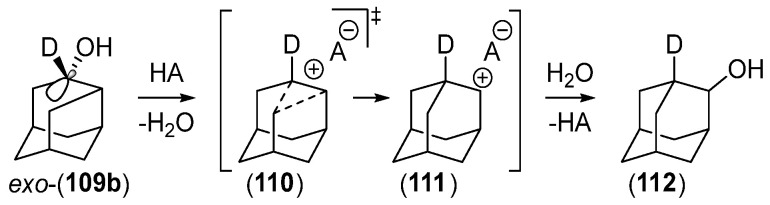
Solvolysis of *exo*-protoadamantan-4-ol-*d*_4_ (**109b**).

The *exo*-isomer **109b** reacts rapidly under acidic conditions to give the corresponding adamantan-2-ol-*d*_1_ (**112**) (Scheme 25). The antibonding orbital lobe of the C–O bond is *syn*-periplanar to the migrating C_2_–C_3_ bond (**110**) and, as a result, ionizes through strong C–C hyperconjugation to the more stable and less strained 2-adamantyl cation (**111**), compared to the secondary but more strained 4-protoadamantyl cation, which is trapped by water. The carbocation has a partially delocalized character [80].

**Scheme 26:**
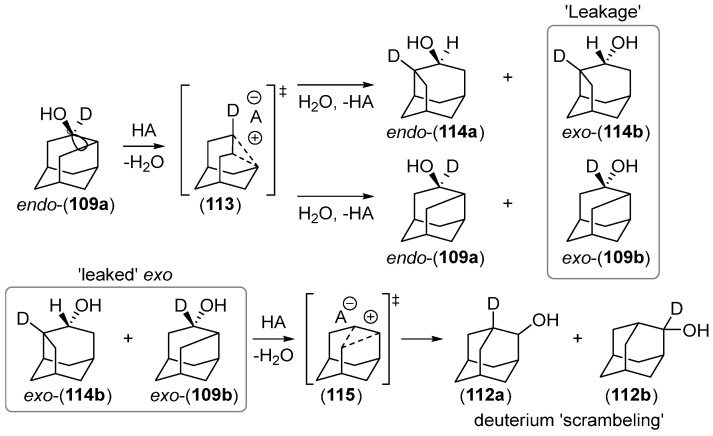
Solvolysis of *endo*-protoadamantan-4-ol-*d*_4_ (**109a**).

The *endo*-isomer (**109a**) reacts much more slowly as the antibonding C–O orbital has an overlap with the C_3_–C_8_ bond, which stabilizes the leaving group through hyperconjugation during ionization (Scheme 26). The result is a more stable, degenerate, bridged (delocalized) carbonium ion **113** that is trapped by water through both *endo* (**109a** and **114a**) and *exo* (**109b** and **114b**) attack. The transition to adamantane occurs through ‘leakage’ to the *exo*-isomer (**109b** and **114b**) that undergoes the same reaction depicted in Scheme 23 (**115**). This is reflected in the reaction kinetics and final product distributions, where the scrambling of the deuterium label (**112a** and **112b**) was observed, in contrast to no scrambling during the solvolysis of the *exo*-isomer (**112a**) [80].

**Scheme 27:**
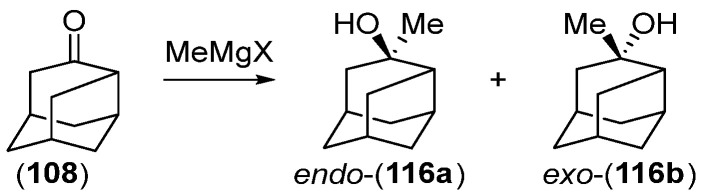
Reaction of Grignard reagent (MeMgX) with ketone **108**.

Upon the introduction of a methyl substituent to the 4-protoadamantyl position, the nature of the transition state changes. The reaction of ketone **108** with Grignard reagent (MeMgX) results in a mixture of *endo*-(**116a**) and *exo*-4-methyl-protoadamantan-4-ol (**116b**) (Scheme 27) [81].

**Scheme 28:**
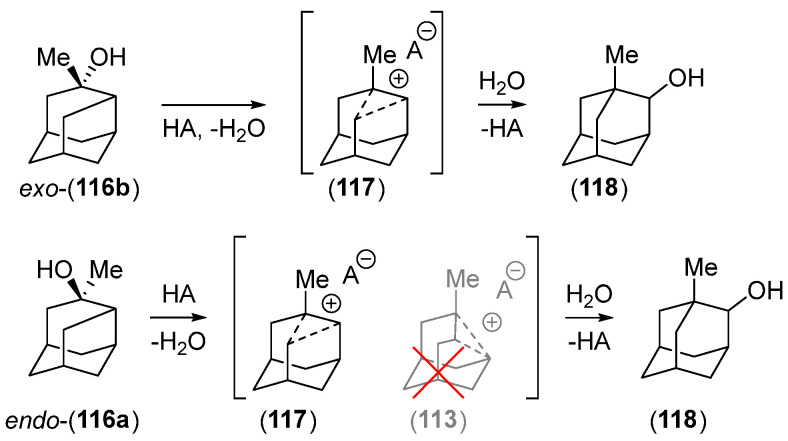
Solvolysis of *endo*-(**116a**) and *exo*-4-methyl-protoadamantan-4-ol (**116b**).

Not only is the rate of solvolysis accelerated by the methyl substituent of the *exo*-isomer **116b** but the transition state is a fully bridged carbonium ion (**117**) as the strained but now third-degree 4-protoadamantyl cation and the less strained second-degree 2-adamantyl cation both have an equal contribution to its stability (Scheme 28).

The effect is also significant during the solvolysis of the *endo*-isomer **116a**. The carbonium ion **113** in Scheme 26 no longer participates as it is not degenerate and is lower in energy. Ionization leads to the same carbonium ion **117** identical to that of the *exo*-isomer (**117**) (Scheme 28). The solvolysis of both *endo*- and *exo*-isomers leads to 1-methyl-adamantan-2-ol (**118**) [82].

**Scheme 29:**
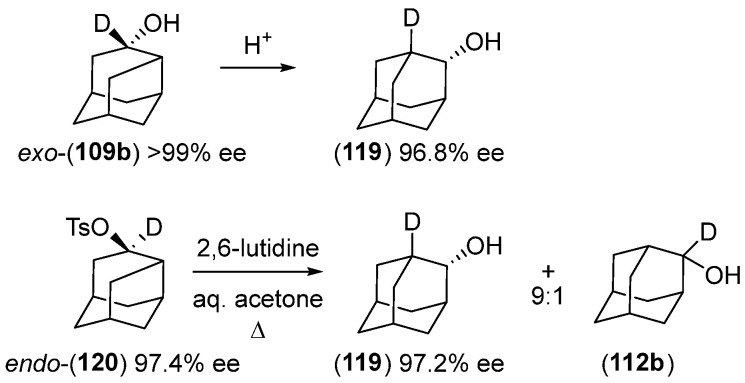
Solvolysis of *exo*-alcohol **109b** and *endo*-tosylate **120** results in retention of enantiopurity.

The peculiar nature of the protoadamantane-adamantane rearrangement is further pronounced by the chirality of the bridged carbocation intermediate. Delocalized electrons occupy one side of the vacant 2-adamantyl p-orbital, forcing a nucleophilic attack to occur from the opposite side with the complete retention of enantiopurity (Scheme 29) [83].

**Scheme 30:**
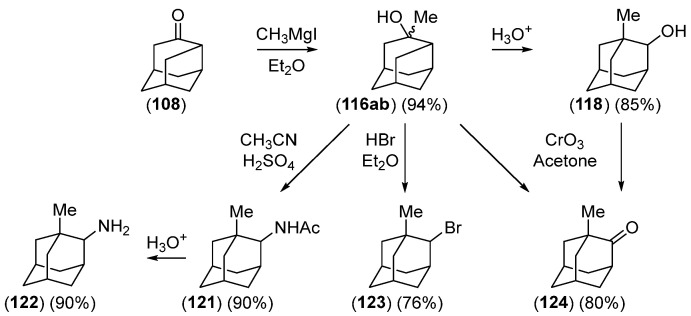
Preparation of various 1,2-disubstituted adamantane derivatives from alcohols **116ab**.

The first efforts to utilize protoadamantan-4-one to synthesize 1,2-disubstituted adamantane derivatives are shown in Scheme 30. Ketone **108** was reacted with Grignard reagent (MeMgI) to produce a mixture of alcohols **116** that were subjected to a standard reaction toolkit. The Ritter reaction gave the corresponding acetamide **121** in a high yield, which can be hydrolyzed to amine **122**. Ethereal hydrobromic acid yielded the 2-bromo derivative **123**. Jones oxidation can be carried out directly on alcohol **116** (80% yield) or on the product of aqueous hydrolysis **118** (80% yield), affording ketone **124** [81].

**Scheme 31:**
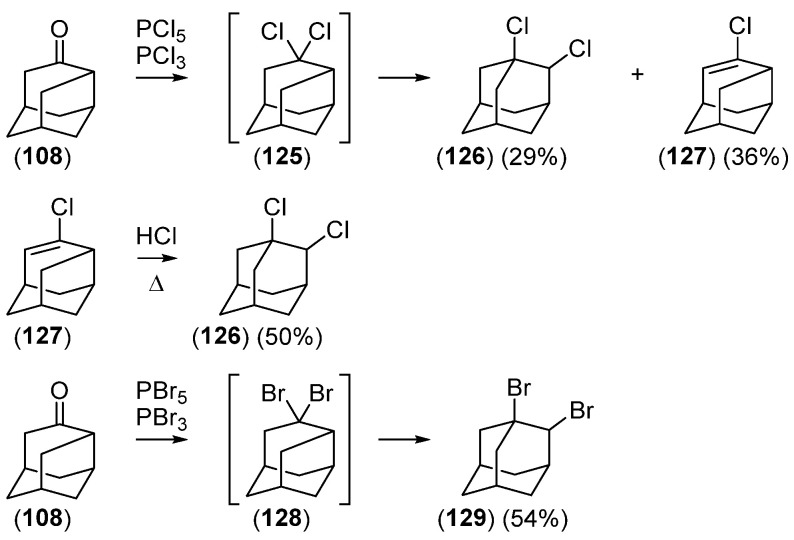
Reaction of ketone **108** with phosphorus tri- and penta-halides.

The reaction of ketone **108** in a mixture of phosphorus tri- and penta-chloride produces a mixture of 1,2-dichloroadamantane **126** and the elimination product **127** from intermediate **125** (Scheme 31), which are easily separable by standard column chromatography on silica gel. Chloro-olefine **127** can be completely transformed to dichloride **126** in hot hydrochloric acid. A similar reaction in phosphorus tri- and penta-bromide led to 1,2-dibromo derivative **129** without the formation of an elimination product [81,84].

**Scheme 32:**
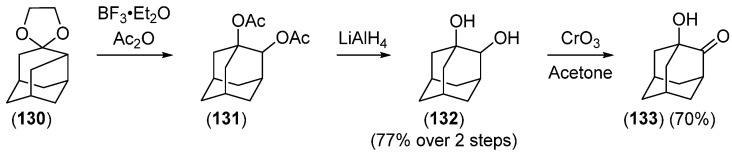
Conversion of protoadamant-4-one (**108**) to adamantane 1,2-diol **132**.

Ethylene acetal **130** (protected ketone **108**) exposed to a boron trifluoride etherate complex in acetic anhydride generates the acylium ion that deprotects and rearranges it to the 1,2-diacetyl ester **131**, which was directly reduced with lithium aluminum hydride, exposing the 1,2-diol **132** in a high yield. Jones oxidation then gives keto-alcohol **133** (Scheme 32) [84].

**Scheme 33:**
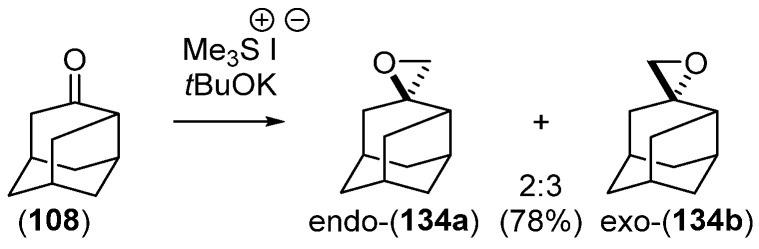
Reaction of ketone **108** with sulfonium methylide gives a mixture of oxiranes **134ab**.

A separable isomeric mixture of oxiranes **134ab** can be obtained by reacting ketone **108** with dimethyl sulfonium methylide (Scheme 33) [85,86].

**Scheme 34:**
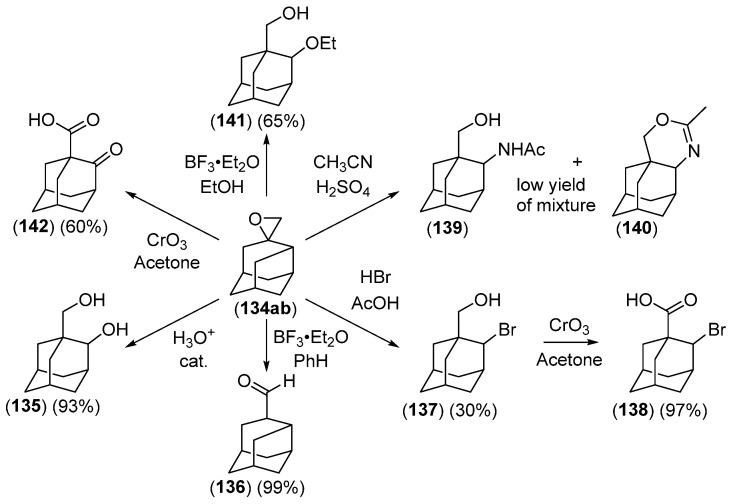
Preparation of various 1,2-disubstituted adamantane derivatives from oxirane **134ab**.

As in previous examples, the mixture of oxiranes **134ab** was subjected to a standard set of reactions (Scheme 34). Catalytic amounts of mineral acids open the oxirane ring, with the carbocation being formed on the inner (more substituted) carbon atom easily rearranging to diol **135**. A Lewis acid-promoted Meinwald rearrangement produced the carbaldehyde **136** instead. Anhydrous hydrobromic acid opened the oxirane to form the 2-bromo derivative **137**, albeit in a lower yield, that was further oxidized with Jones reagent to 2-bromoadamantane-1-carboxylic acid **138**. Ritter reaction conditions gave a complex mixture of products with only a small amount of the desired acetamide **139** accompanied by the cyclic side product **140**. Lewis acid opening in ethanol as a solvent gave ethyl ether **141**. Finally, Jones oxidation directly transformed oxirane **134ab** to the β-keto-carboxylic acid **142** [86].

**Scheme 35:**
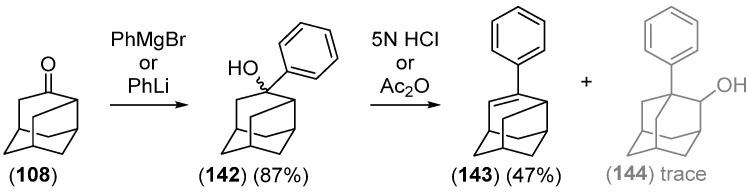
Attempted rearrangement of alcohols **142**.

The reaction of ketone **108** with phenyl magnesium bromide or phenyl lithium resulted in a mixture of alcohols **142** (Scheme 35). Attempts to obtain the rearranged alcohols **144** were rather unsuccessful at first (see Scheme 39) when performing the reaction in hot 5N HCl or acetic anhydride, giving predominantly the elimination product **143** [86].

**Scheme 36:**
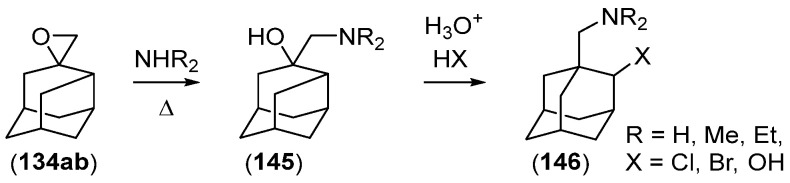
Opening of oxirane **134ab** under basic conditions followed by acid-promoted rearrangement gave amino-alcohols **146**.

Oxirane **134ab** was also successfully opened under basic conditions, forming the expected amino-alcohols **145** (Scheme 36). The exposure of **145** to aqueous acids gave the corresponding rearranged adamantyl amino-alcohols **146** (X = OH), whereas anhydrous halo-acids gave the 2-halo derivatives **146** (X = Cl, Br) in good to high yields [87].

**Scheme 37:**
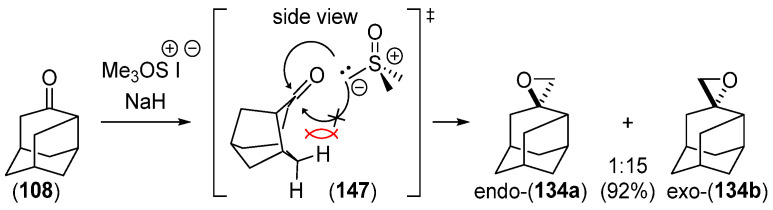
Epoxidation of ketone **108** using sulfoxonium methylide leads to the *exo*-isomer **134b** as the major component.

The epoxidation of ketone **108** using dimethyl sulfoxonium methylide instead changes the product distribution of oxiranes **134ab**, shifting the ratio to *exo*:*endo* = 15:1, likely due to the reagent’s bulk, favoring the kinetic *exo*-attack (**147**) over the *endo*-attack, being hindered by the C_7_ hydrogens (Scheme 37) [88].

**Scheme 38:**
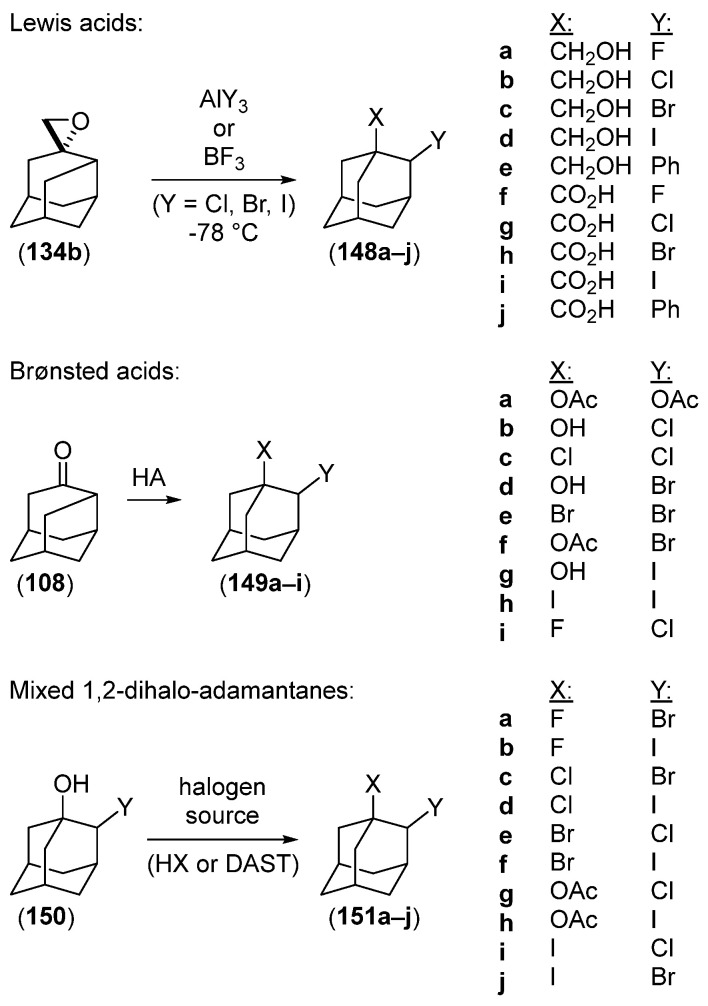
Synthesis of various 1,2-disubstituted derivatives from *exo*-**134b**.

Continuing with the *exo*-oxirane **134b** in Scheme 38, the authors prepared a large amount of 1,2-disubstituted adamantane derivatives using various methods.

Ring opening and rearrangement were triggered by the use of aluminum halides or boron trifluoride etherate at cryogenic temperatures to gain a wide range of 2-halo-adamantylmethylene alcohols **148a–d** that were further oxidized by Jones reagent to their carboxylic acid derivatives **148f–j**. Carrying out the reaction in benzene with an excess of Lewis acid resulted in the formation of a 2-halo derivative that further reacted via the Friedel–Crafts reaction, giving the 2-phenyl-substituted compounds **148e** and **148j**. The yields reported were all above 80%.

Optimized procedures for the preparation of 2-halo-adamantanols **149a,b,d,f,g** and 1,2-dihalo-adamantanes **149c,e,h,i** on exposure to halo-acids at various conditions were also carried out.

The prepared 2-halo-adamantanols **149a,b,d,f,g** were further reacted to prepare a variety of mixed 1,2-dihalo-adamantanes **151a–f,i,j** by displacement of the 1-hydroxy group using corresponding halogen acids (dimethylaminosulfur trifluoride = DAST in the case of X = F) with yields above 60% [88].

**Scheme 39:**
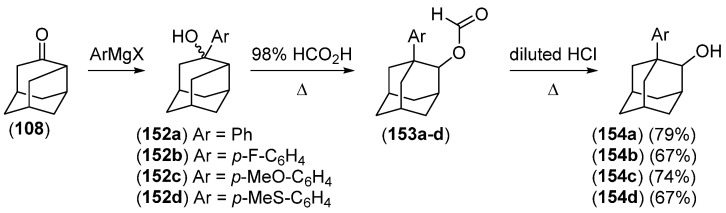
Preparation of 1-aryl-adamantan-2-ols **154a–d**.

Following previously unsuccessful attempts (Scheme 35), the reaction of ketone **108** with aryl Grignard reagents gave an isomeric mixture of alcohols **152a–d** that were subsequently refluxed in formic acid, rearranging to the formic esters **153a–d** (Scheme 39). These were hydrolyzed in hot dilute aqueous HCl, successfully producing the desired 1-aryl-adamantan-2-ols **154a–d** in good yields. Although the products were not reported, the authors claimed to have prepared 1-aryl-2-haloadamantanes in good yields when exposing the alcohols **152** to hydrogen chloride/bromide, seemingly without the issue of the elimination products that were described in Scheme 35 [89].

**Scheme 40:**
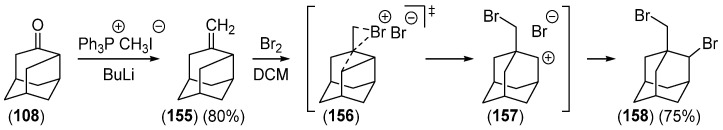
Wittig reaction with ketone **108** followed by bromine-initiated rearrangement of olefine **155** to form dibromide **158**.

A Wittig reaction with ketone **108** was demonstrated to synthesize the olefine **155** (Scheme 40). Exposure to bromine rearranged the olefine **155** to the dibromide **158**. The bromonium ion **156** results in positive charge development on the inner, more substituted carbon atom, triggering a fast intramolecular rearrangement to the 2-adamantyl cation **157** that is trapped by the bromide anion [90].

**Scheme 41:**
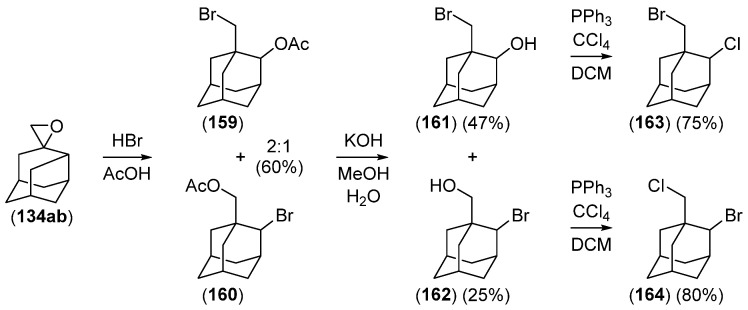
Preparation of mixed 2-halo-1-halomethylene adamantanes **163** and **164**.

The opening of oxirane **134ab** with 33% hydrobromic acid in acetic acid gave a mixture of acetyl esters **159** and **160** that were further hydrolyzed to their alcohols **161** and **162**, with a finishing Appel reaction executing the oxygen-halogen exchange, producing mixed dihalo compounds **163** and **164** (Scheme 41) [90,91].

**Scheme 42:**
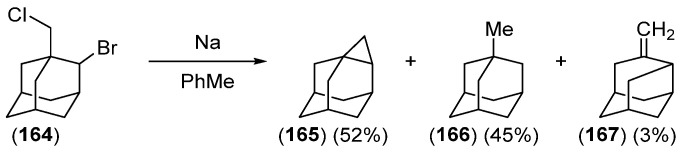
Preparation of 1,2-methanoadamantane **165** using Wurtz coupling.

The prepared 2-halo-1-halomethylene adamantanes **158**, **163**, and **164** (Scheme 40 and Scheme 41) were used for the optimization of the reaction conditions for the synthesis of highly strained and reactive 1,2-methanoadamantane **165** using Wurtz coupling (Scheme 42). The cyclopropyl ring of **165** possesses a twisted σ-bond, making it extremely reactive towards weak acids (water, methanol), leading to expansion to homoadamantane, but it is inert towards bases. The compound was purified by vacuum distillation and its structure was determined using spectroscopic analyses. The best results were obtained with starting material **164**, metallic sodium in toluene at reflux. Product distributions were heavily dependent on the solvents used. Alkyl lithium reagents failed to generate cyclopropane **165** through trans-halogenation [90,91].

**Scheme 43:**
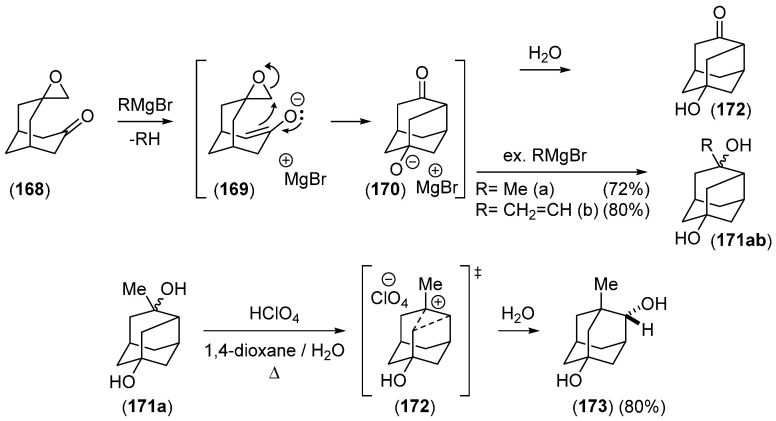
Synthesis of bridgehead substituted protoadamantyl derivatives **171ab** and **172**. Rearrangement of diol **171a** to 1,3,4-trisubstituted adamantane **173**.

Epoxy ketone derivative **168** of the ketoolefine **65** (Scheme 15) was reacted with Grignard reagent to produce the 4-alkyl-4-hydroxyprotoadamantan-1-ols **171ab** in high yields (Scheme 43). Using equimolar amounts of the Grignard reagents resulted in the enolization of ketone **168**, followed by transannular cyclization (**169**) opening the epoxide from the less hindered side (basic conditions), forming the protoadamantane cage **170**. Upon aqueous work-up, the corresponding keto-alcohol **172** was obtained. Using an excess of the Grignard reagent leads to the further addition to keto-alcohol **172**, giving isomeric mixtures of *endo*- and *exo*-diols **171ab**. Transmetalation of the Grignard reagent to CeCl_3_ gave oxaadamantane derivatives (oxygen atom embedded in the adamantane framework). The cyclization of starting material **168** was first demonstrated using potassium *tert*-butoxide as a base [54]. The prepared racemic diol **171a** was rearranged with perchloric acid in hot aqueous dioxane to afford the racemic 1,3,4-trisubstituted adamantane diol **173** in 80% [92].

**Scheme 44:**
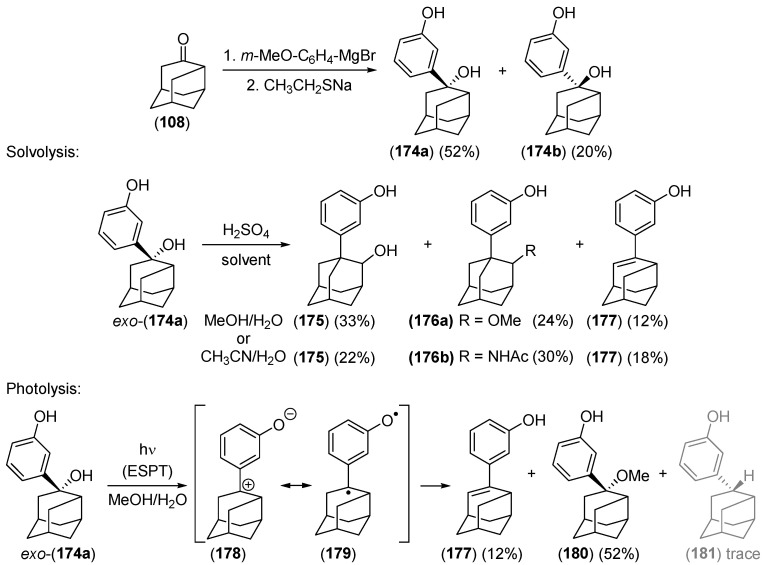
Solvolysis and photolysis of diol **174a**.

A study to probe the difference in reactivity of zwitterionic *m*-quinone methides, generated by photo-induced excited-state proton transfer (ESPT), and carbocations generated thermally in acidic media was carried out (Scheme 44). Ketone **108** was reacted with an aryl Grignard reagent, followed by cleavage of the methyl ether with sodium thiolate to give aryl alcohols **174ab**, which were separated by column chromatography into individual isomers. The *exo*-isomer **174a** was exposed to sulfuric acid in different solvent systems, which impacted the product distribution of the rearranged diol **175**, the rearranged substitution products **176ab**, and the elimination product **177**. The reaction carried out in 3:1 MeOH:H_2_O produced the diol **175** as the major product, followed by the methoxy adamantane **176a**, with small amounts of elimination product **177**. In 2:1 CH_3_CN:H_2_O, the Ritter reaction produced the acetamide **176b** as the major product in 30%, with the alcohol **175** and elimination product **177** in similar quantities.

Photolysis in methanol (254 nm) gave the unrearranged substitution product **180** as the major component (52%) and 12% of elimination product **177** without rearranged products (Scheme 42). The zwitterion **178** stabilizes the positive charge of the benzylic cation through conjugation with the phenoxide anion, diminishing its carbonium (bridged) character typical for the protoadamantyl systems, prohibiting the rearrangement to adamantane, leading to substitution products instead. The protonated phenol species during solvolysis still maintains its delocalized character, giving rearranged products [93].

**Scheme 45:**
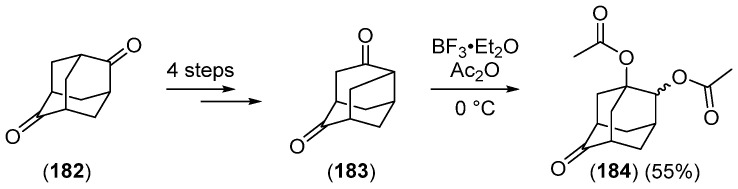
Transformation of di-one **182** to compound **184**.

The inability of adamantane di-one **182** to undergo further bridgehead oxidation prompted the authors to find an alternative procedure to their desired compound (Scheme 45). Through bromination, isomerization, and halogen-oxygen exchange with silver salts, the di-one **182** was converted into the protoadamantyl di-one **183** in four steps. A boron trifluoride etherate complex in acetic anhydride at a low temperature led to the acylative opening of the protoadamantane di-one **183** and afforded the diacetate **184** (the triol precursor) in a 55% yield [94].

**Scheme 46:**
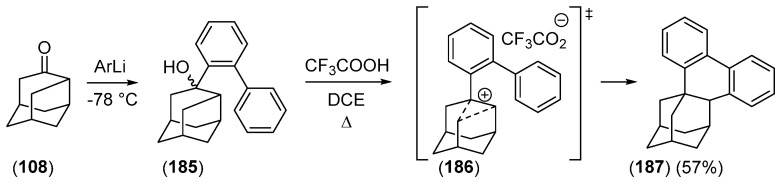
Synthesis of 1,2-annulated adamantane arene **187**; using the same approach, various annulated arenes and heterocycles were synthesized.

The most recent application of the protoadamantane-adamantane rearrangement was demonstrated in the synthesis of 1,2-annulated adamantane arenes (Scheme 46). *Ortho*-lithiated biphenyl, formed from its bromide through lithium-halogen exchange, was combined with ketone **108** to give an isomeric mixture of alcohols **185** that were slightly air-unstable and carried to the next step without purification. Reflux in 1,2-dichloroethane with trifluoroacetic acid formed the bridged cation **186** followed by Friedel–Crafts alkylation to the 2-adamantyl position, giving the adamantane annulated biphenyl **187** in 57% over two steps. Sulfuric acid and BF_3_·Et_2_O were ineffective. The authors managed to showcase their method with a number of various polyaromatic compounds, including heterocycles and halogenated arenes. The motivation behind the work was the synthesis of peripherally modified tunable arenes with improved physical and chemical properties as new materials [95].

The appearance of adamantane, namely adamantyl amines, in the field of medicinal chemistry began during the growth of its chemistry in the 1960s with the success of simple 1-aminoadamantane (Amantadine) used as an antiviral agent against Influenza A and greatly reducing the effects of Parkinson’s disease. Since then, a myriad of adamantane-containing drug molecules with various therapeutic targets have emerged, with more being developed [7].

**Scheme 47:**
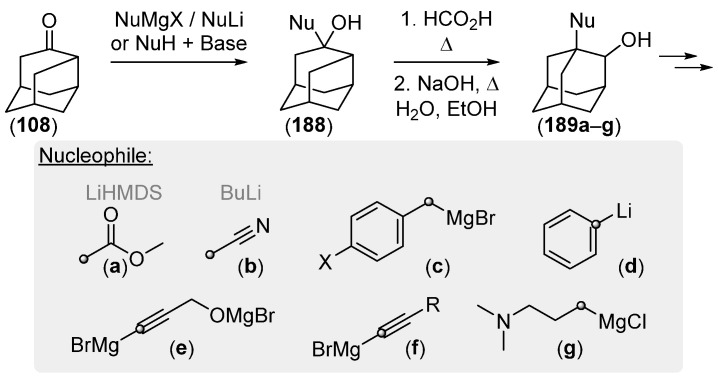
Synthesis of biologically active compounds through the protoadamantane-adamantane rearrangement starting from ketone **108** reacted with various nucleophiles.

The protoadamantane-adamantane rearrangement is one of the key transformations employed in the synthesis of 1,2-disubstituted adamantyl amines. Many authors have conducted extensive work describing a plethora of examples of connectivity between ketone **108** and various nucleophiles (Scheme 47), followed by the rearrangement to adamantane and further syntheses to their target compounds, to analyze their biological activities [76,96,97,98,99,100,101,102]. For compounds, see **189a,e** [76]; **189b,d,g** [99]; **189c** [98]; **189f** [96] (Scheme 47).

#### 2.3.2. The Noradamantane Route

**Scheme 48:**
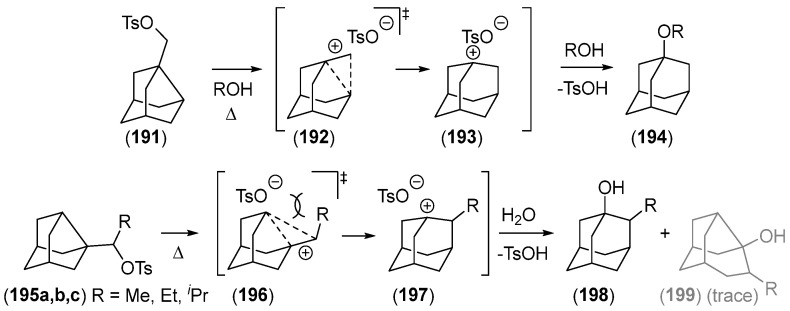
Mechanism of the noradamantane-adamantane rearrangement.

In parallel with studies on protoadamantane derivatives, the rearrangement of noradamantane to adamantane has been described [103]. Solvolytic studies of the neopentyl-like tosyl ester **191** have pointed out the rapidity of the process, being 17,000 times faster compared to that of neopentyl tosylate itself (Scheme 48) [104]. The driving force is the release of the strain and rearrangement to the highly stable 1-adamantyl cation **193** [105]. It is worth noting that hydride shifts between positions (carbons) 1 and 2 are prohibited due to geometric constraints [106]. If the migration of a substituent from position 2 to 1 occurs, then it proceeds through a series of intramolecular C–C bond rearrangements [107].

**Scheme 49:**
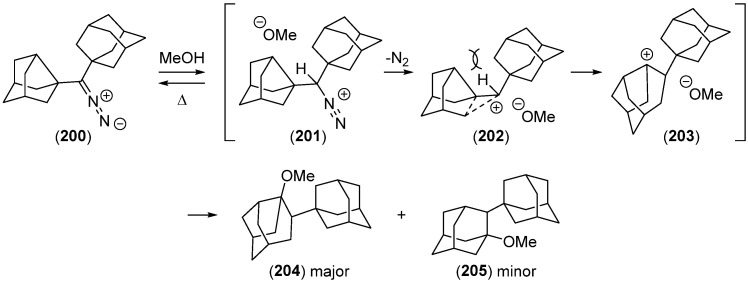
Diazo compound **200** rearranges through the migration of the C_2_–C_3_ bond, forming **204** as the major product.

As was the case with protoadamantane, the rearrangement proceeds through a highly delocalized transition state **192** with the strong hyperconjugation of the migrating C_3_–C_7_ bond to the antibonding orbital of the leaving group during ionization. In other words, the rearrangement has a strong S_N_2 character. An increase in the size of the α-substituent (**195abc**) retards the rate of rearrangement by steric hindrance of the approaching C_3_–C_7_ bond with protoadamantyl side products **199**, resulting from the migration of the C_2_–C_3_ bond instead (Scheme 48 and Scheme 49) [105].

Under kinetic control with adamantane as the α-substituent (Scheme 49), a set of conditions that favored the migration of the C_2_–C_3_ bond were found. The diazo compound **200** generates the carbocation **202** upon the loss of nitrogen and rearranges through the unhindered C_2_–C_3_ bond to the 3-protoadamantyl cation **203**. The protoadamantyl product **204** was formed as the major component, with the adamantyl derivative **205** as the minor one [108].

**Scheme 50:**
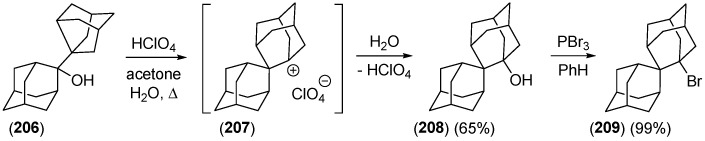
Acid-promoted rearrangement of alcohol **206** to 1-hydroxy[1]diadamantane **208**.

The noradamantane-adamantane expansion was utilized in the synthesis of spirocompounds **208** and **209** (Scheme 50). The exposure of alcohol **206** to perchloric acid in hot aqueous acetone gave the rearranged 1-hydroxy[1]diadamantane **208** in a 65% yield. Oxygen-bromine exchange with phosphorus tribromide in anhydrous benzene provided the bromide **209** in a quantitative yield [109].

**Scheme 51:**
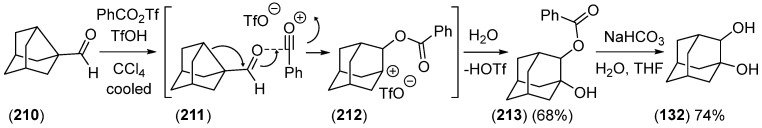
Ring expansion of carbaldehyde **210** to benzoyl ester **213** followed by hydrolysis to diol **132**.

As part of the substrate scope for the ring expansion of polycyclic hydrocarbons from their carbaldehydes to expanded diols, the noradamantyl carbaldehyde **210** (Scheme 51) was added to a solution containing an in situ generated phenyl acylium ion (**211**) that promoted the ring expansion to intermediate **212**, which was quenched by an aqueous work-up to give benzoyl ester **213** (68%). Basic hydrolysis released the 1,2-adamantane diol **132** (74%) [110].

**Scheme 52:**
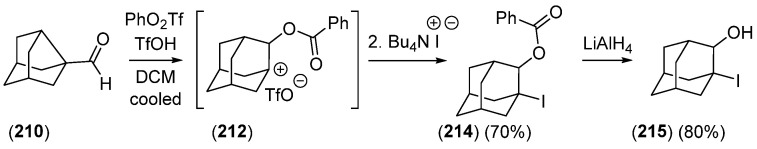
Ring expansion of carbaldehyde **210** with addition of Bu_4_N^+^I^−^ forms iodo-ester **214**.

The addition of tetrabutylammonium iodide to the acylative ring expansion of carbaldehyde **210** trapped the intermediate carbocation **212** with iodide, forming the iodo-ester **214** (70%) (Scheme 52). Lithium aluminum hydride reduction of the ester gave alcohol **215** (80%) [111].

**Scheme 53:**
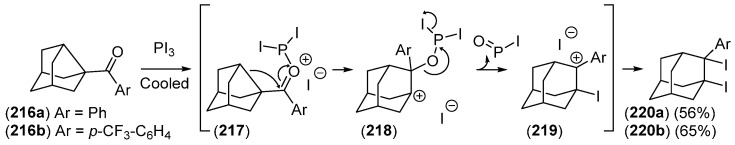
Phosphorus promoted rearrangement of phenones **216ab** to diiodides **220ab**.

Ring expansion of the noradamantyl phenones **216ab** was achieved by their addition to a solution of red phosphorus and iodine (PI_3_) (Scheme 53). After the addition of phosphorus to the carbonyl oxygen, the oxonium ion **217** triggers rearrangement with the released iodide trapping the 1-adamantyl cation **218**. The formation of the second phosphorus-oxygen bond drives the C–O bond cleavage, forming a stable benzylic carbocation **219** that is trapped by a second iodide to give the diiodo derivatives **220ab** [112].

**Scheme 54:**
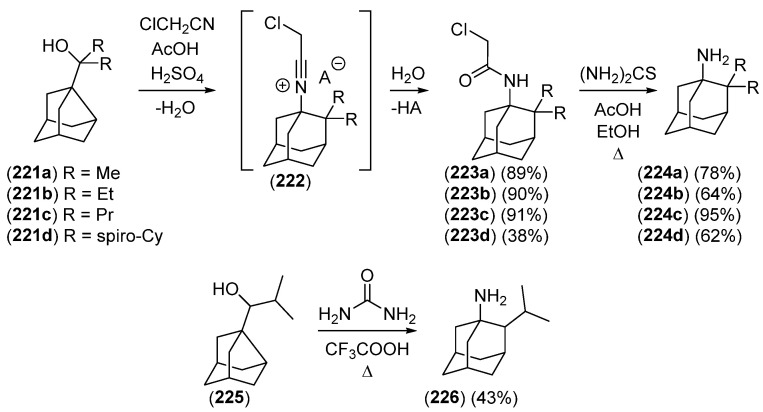
Ritter reaction of alcohols **221a–d** followed by hydrolysis produced amines **224a–d**.

Similarly to protoadamantane, the noradamantane-adamantane rearrangement can be utilized in the synthesis of 1,2-disubstituted adamantyl amines to combat the Influenza A virus (Scheme 54). A variety of alkyl-substituted (3-noradamantyl)methylene alcohols **221a–d** were subjected to the Ritter reaction, opting for chloroacetonitrile for ease of the following hydrolysis step. The formed 1-adamantyl carbocation was trapped with chloroacetonitrile (**222**) with the addition of water, giving the corresponding acetamides **223a–d** in high yields, which were hydrolyzed with thiourea and acetic acid, releasing the free amines **224a–d**.

Additionally, the authors managed to prepare amine **226** in a one-pot procedure by refluxing alcohol **225** in trifluoroacetic acid with an excess of urea. However, the yields were reportedly lower than the combined yields of the general two-step procedure [113].

**Scheme 55:**
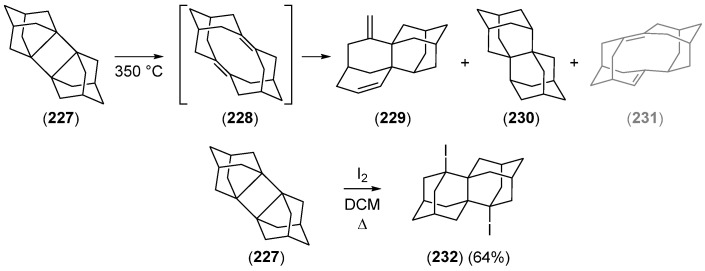
Thermolysis and iodination of cyclobutane derivative **227**.

During the work with cyclobutane derivative **227** (Scheme 55), the authors carried out computational and experimental studies on its thermolysis for future research. Short reaction times at high temperatures in vacuum gave the ring-opened diene **228**. Longer exposure gave a mixture of products with adamantane scaffolds **229**, **230** and proposed **231**. Upon mechanistic investigation, thermolysis was carried out in the presence of iodine to trap potential radical intermediates. Upon lowering the temperature, the diiodide **232** was formed as the major product. The preparation of diiodide **232** for synthetic purposes was performed by heating cyclobutane **227** with iodine in dichloromethane under reflux [114].

**Scheme 56:**
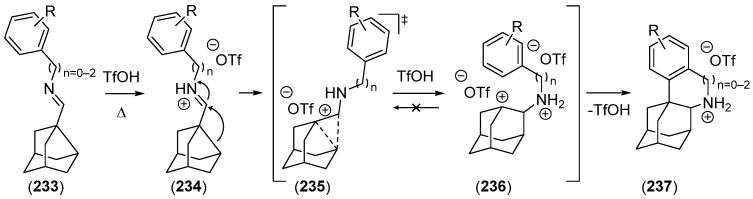
Synthesis of 1,2-annulated aza-heterocyclic diamondoid derivatives **237**. Using the same approach, various 5-, 6-, and 7-membered annulated heterocycles were prepared.

The last example (Scheme 56) links the nucleophilic noradamantane-adamantane rearrangement to an intramolecular electrophilic aromatic substitution reaction (Friedel–Crafts reaction with the 1-adamantyl cationic intermediate as an electrophile) to prepare 1,2-annulated aza-heterocyclic adamantane derivatives. The starting materials **233** used were prepared by condensation of the noradamantyl carbaldehyde **210** with anilines, benzyl amines, and phenylethyl amines. Reaction with triflic acid in 1,2-dichlorobenzene at 140 °C initially forms the iminium salt **234**. The presence of triflic acid in excess traps the intermediate **236** of the reversible rearrangement by protonating the amine, disabling its return to the more stable iminium salt **234**. The non-nucleophilic anion-stabilized 1-adamantyl cation **236** is captured by intramolecular Friedel–Crafts alkylation with the aryl moiety, giving the annulated products **237**. The authors successfully applied the method, creating five-, six-, and seven-membered rings with variously substituted aryls and alkyl substituted adamantanes. Intermolecular Friedel–Crafts reactions were also demonstrated when using the arenes as a solvent or in high excess.

Although the process is catalytic, full conversion was achieved with two equivalents of acid. Weaker Bronsted acids, such as HCl or CF_3_COOH, and Lewis acids (Cu(OTf)_2_) did not trigger the reaction. Lowering the temperature decreased the yield significantly, with no reaction being observed at 80 °C due to the low solubility of TfOH in 1,2-dichlorobenzene [115].

## 3. Conclusions

Progress in chemistry is often based on the availability of simple building blocks, which can be used directly for further purposes. The 1,2-disubstituted adamantane compounds are such a class of compounds. The synthesis of these derivatives relies either on C–H functionalization methods or the construction of the cage framework. We hope that this review will inspire future researchers to find new methods that can be applied for the synthesis of adamantane derivatives or bridged cycloalkanes in general.

## Data Availability

Not applicable.
